# Effect of *Crocus sativus* (Saffron) Intake on Top of Standard Treatment, on Disease Outcomes and Comorbidities in Patients with Rheumatic Diseases: Synthesis without Meta-Analysis (SWiM) and Level of Adherence to the CONSORT Statement for Randomized Controlled Trials Delivering Herbal Medicine Interventions

**DOI:** 10.3390/nu13124274

**Published:** 2021-11-27

**Authors:** Sotirios G. Tsiogkas, Maria G. Grammatikopoulou, Konstantinos Gkiouras, Efterpi Zafiriou, Iordanis Papadopoulos, Christos Liaskos, Efthimios Dardiotis, Lazaros I. Sakkas, Dimitrios P. Bogdanos

**Affiliations:** 1 Department of Rheumatology and Clinical Immunology, Faculty of Medicine, School of Health Sciences, University of Thessaly, Biopolis, GR-41334 Larissa, Greece; stsiogkas@gmail.com (S.G.T.); mariagram@auth.gr (M.G.G.); kostasgkiouras@hotmail.com (K.G.); liaskosch@uth.gr (C.L.); lsakkas@med.uth.gr (L.I.S.); 2 Faculty of Medicine, School of Health Sciences, Aristotle University of Thessaloniki, GR-54124 Thessaloniki, Greece; 3 Department of Nutritional Sciences & Dietetics, Faculty of Health Sciences, Alexander Campus, International Hellenic University, GR-57400 Thessaloniki, Greece; driordanis@yahoo.gr; 4 Department of Dermatology, Faculty of Medicine, School of Health Sciences, University of Thessaly, Biopolis, GR-41334 Larissa, Greece; zafevi@med.uth.gr; 5 Department of Neurology, Faculty of Medicine, School of Health Sciences, University of Thessaly, Biopolis, GR-41334 Larissa, Greece; edar@med.uth.gr

**Keywords:** crocin, crocetin, safranal, effect direction plot, complementary and alternative medicine, medicinal plant, CAM, herbal medicine, TNF-α, qualitative synthesis, medicinal plant, dietary supplements

## Abstract

Rheumatic diseases (RDs) are often complicated by chronic symptoms and frequent side-effects associated with their treatment. Saffron, a spice derived from the *Crocus sativus L*. flower, is a popular complementary and alternative medicine among patients with RDs. The present systematic review aimed to summarize the available evidence regarding the efficacy of supplementation with saffron on disease outcomes and comorbidities in patients with RD diagnoses. PubMed, CENTRAL, clinicaltrials.gov and the grey literature were searched until October 2021, and relevant randomized controlled trials (RCTs) were screened for eligibility using Rayyan. Risk of bias was assessed using the Cochrane’s Risk of Bias-2.0 (RoB) tool. A synthesis without meta-analysis (SWiM) was performed by vote counting and an effect direction plot was created. Out of 125 reports, seven fulfilled the eligibility criteria belonging to five RCTs and were included in the SWiM. The RCTs involved patients with rheumatoid arthritis, osteoarthritis and fibromyalgia, and evaluated outcomes related to pain, disease activity, depression, immune response, inflammation, oxidative stress, health, fatigue and functional ability. The majority of trials demonstrated some concerns regarding overall bias. Moreover, the majority of trialists failed to adhere to the formula elaborations suggested by the CONSORT statement for RCTs incorporating herbal medicine interventions. Standardization of herbal medicine confirms its identity, purity and quality; however, the majority of trials failed to adhere to these guidelines. Due to the great heterogeneity and the lack of important information regarding the standardization and content of herbal interventions, it appears that the evidence is not enough to secure a direction of effect for any of the examined outcomes.

## 1. Introduction

Rheumatic and musculoskeletal diseases have the highest population impact across all adverse health outcomes, including greater disability-adjusted life years (DALY) [1,2]. Due to the chronic nature of these conditions and the frequent side-effects associated with their treatment, patients often resort to complementary and alternative medicines (CAMs), in search of “less toxic” therapies [3,4].

Garlic, ginger, curcumin, cinnamon, or saffron are a few of the most popular CAMs used in rheumatic diseases (RDs) [5,6,7]. Saffron, in particular, is the dried stigma of the flowers of *Crocus sativus L*. (family *Iridaceae*), cultivated mainly in Southern Europe, India and Iran, and is considered as one of the most expensive culinary spices globally [8]. The medicinal properties of saffron and its constituents (safranal, crocin, and crocetin) include anti-inflammatory, antioxidant, analgesic, antihypertensive, hypolipidemic, antitussive, anticonvulsant, antidepressant, anxiolytic, anticancer, and antinociceptive characteristics [9,10,11,12,13,14,15]. Nevertheless, although saffron supplementation has been tested in patients with various RDs employing a randomized controlled trial (RCT) design, we have insufficient evidence regarding its efficacy, as no systematic reviews have attempted to synthetize these data in order to aid in the formulation of recommendations.

A common issue in CAM research, however, is the lack of standardization of the administered products, often resulting in an inability to reproduce the findings and understand which active ingredients may in fact propel the observed outcomes. The standardization of herbal medicine confirms its identity, purity and quality, and for this, relevant trials ought to disclose information regarding formula elaborations [16]. This information is required to judge the internal validity, external validity, and reproducibility of the administered interventions [17,18].

The aim of the present systematic review was to evaluate the efficacy of saffron oral nutrient supplementation (ONS) on top of standard treatment, on disease outcomes and comorbidities in patients with RDs and evaluate the quality of these trials.

## 2. Materials and Methods

### 2.1. Systematic Review Protocol and PIO

The Preferred Reporting Items for Systematic reviews and Meta-Analyses (PRISMA) [19] and the Synthesis Without Meta-analysis (SWiM) extension [20] were used for the presentation of the present review. The study’s protocol was published at the center for open science framework (OSF) (https://bit.ly/3pHeSa7, accessed on 26 November 2021).

The PICO of the study’s research question is detailed in Table 1.

### 2.2. Search Strategy and Algorithm

**Figure 1:**
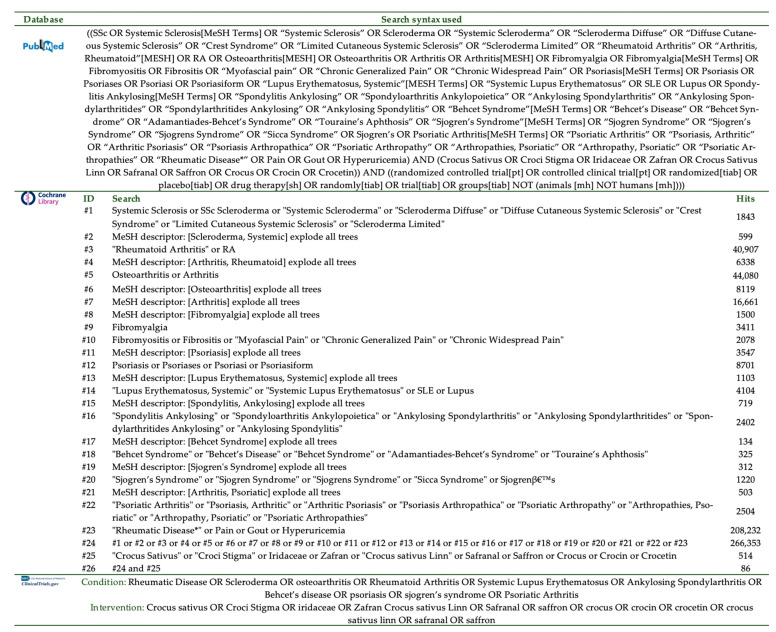
Search syntax used in the databases.

Studies related to the research question were identified through PubMed, the Cochrane Central Register of Controlled Trials (CENTRAL), clinicaltrials.gov and grey literature searches from inception until October 2021 by three independent reviewers (S.G.T., M.G.G. and K.G.). Any disagreement between reviewers was resolved by a senior researcher (D.P.B.). The search syntax used in each database is presented in Figure 1.

Rayyan [21], a web and mobile application for conducting systematic reviews, was used to scan and identify all studies fulfilling the study’s criteria. All identified references were imported into Rayyan using reference manager software, and duplicate entries were excluded.

Combinations of relevant keywords were used to identify relevant RCTs in the literature. The keywords used included (Crocus sativus), (saffron), (crocin), (crocetin), (safranal), (rheumatoid arthritis), (scleroderma), (fibromyalgia), (Behçet’s syndrome), (osteoarthritis), (hyperuricemia), (gout), (ankylosing spondylitis), (psoriatic arthritis), (psoriasis), (psoriatic plaque), (spondylarthritis), (systemic lupus erythematosus), (lupus), (SLE), (Sjogren’s syndrome), (systemic sclerosis), and (rheumatic disease*).

Although not belonging to the RDs, osteoarthritis (OA) was also included in the search strategy, since many patients with RA are often misdiagnosed with OA and vice versa [22].

### 2.3. Inclusion and Exclusion Criteria

Studies were included in the synthesis when they (1) had an RCT design, (2) were parallel or cross-over, (3) used an active *per os* intervention with saffron in any form (tabs, caps, powder, syrup, sachets, tea), (4) were conducted in patients with a RD diagnosis (or osteoarthritis), (5) examined any age group, and (6) used a placebo or any other intervention as a comparator (comparative effectiveness studies).

Exclusion criteria involved (1) all other study designs (non-interventional) including those lacking a comparator arm, (2) studies not including patients with RDs, (3) or using interventions lacking saffron, (4) interventions with curcumin, and (5) published protocols without published results, as well as (6) studies on animals or preclinical studies.

Special caution was taken not to include RCTs investigating the effects of curcumin, which is also known as “Indian saffron” [23].

### 2.4. Outcomes of Interest

Outcomes of interest involved any specific index/score for RDs, including disease activity scores, pain, inflammation markers, antioxidant and oxidative stress status, anxiety, depression, quality of life (QoL), health assessment, immune response indicators, etc.

### 2.5. Risk of Bias

Eligible studies were independently assessed for bias using the Cochrane’s revised Risk of Bias (RoB) tool 2.0 [24] by two authors (K.G. and M.G.G.). Judgments were made if there was a low risk, some concerns or high risk of bias in terms of the randomization process, deviations from intended interventions, missing outcome data, measurement of the outcomes, selection of the reported results and the final assessment regarding the overall bias.

### 2.6. Data Extraction

Two independent researchers (M.G.G. and K.G.) extracted data in Excel spreadsheets. Information regarding the sample (size, RD diagnosis, age, % female), recruitment, country of origin, funding, design and methodology (randomization particularities, masking, etc.), intervention (standardization particularities and dosage) and comparator arms, outcomes of interest, drop-outs, adverse events, presented analysis, and general results was extracted for all studies.

### 2.7. Data Synthesis

At least three RCTs investigating the same outcome for each RD were required for an effective data synthesis. Since a meta-analysis was not feasible, vote counting was applied, based on the direction of effect (mean differences) for each outcome [25] in order to accompany the narrative synthesis [26].

The methodological characteristics of each study (RD diagnosis, overall risk of bias, etc.) were used to assess heterogeneity, according to the Cochrane Handbook [26] and the SWiM guidelines [20].

## 3. Results

### 3.1. Search Results

**Figure 2:**
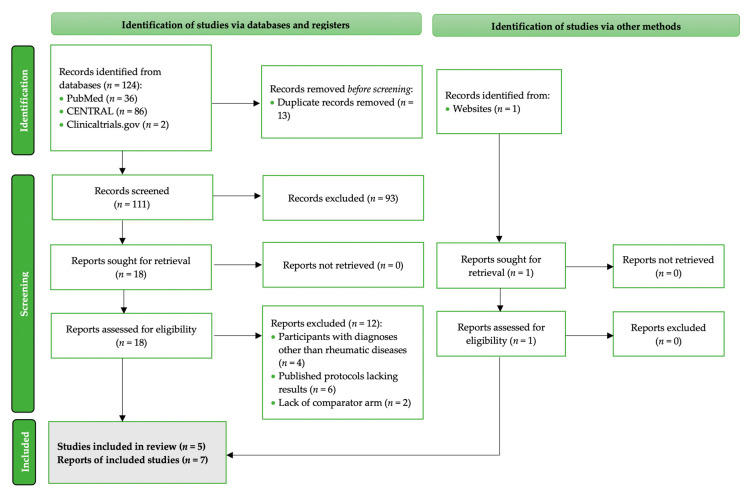
PRISMA [19] flow diagram of the studies’ selection process.

Out of 139 studies screened in total, five RCTs and seven publications (two studies with duplicate publications) [27,28,29,30,31,32,33] fulfilled the protocol’s criteria and were included in the systematic review. Figure 2 details the PRISMA 2020 flow diagram of the study selection process [19].

### 3.2. Characteristics of RCTs with Saffron Interventions in Patients with Rheumatic Diseases

#### 3.2.1. RD Diagnoses

**Figure 3:**
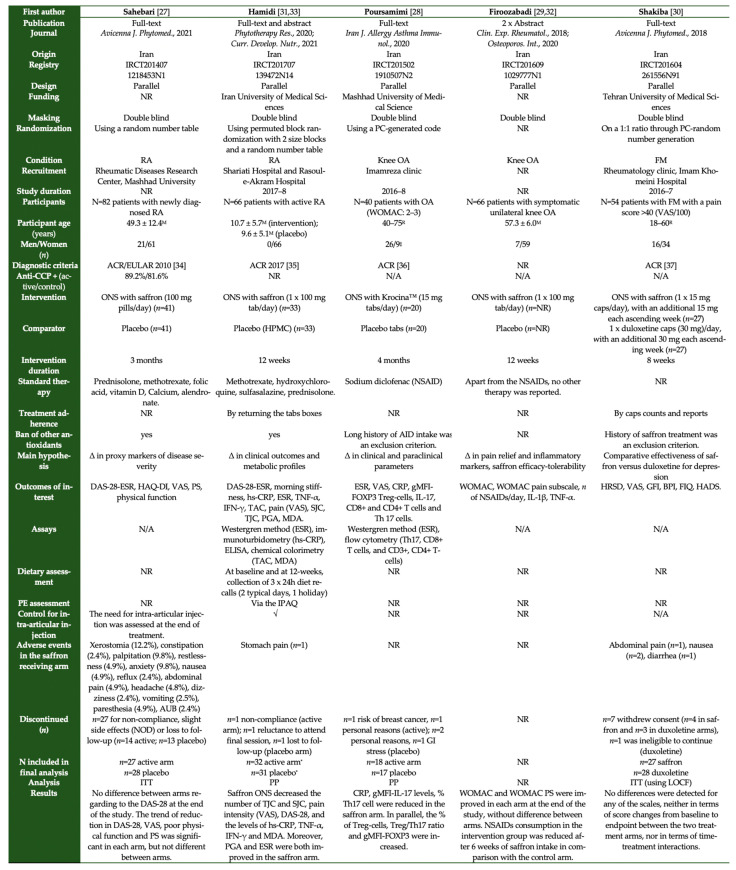
Characteristics of the parallel RCTs evaluating interventions with saffron in patients with RA, OA, or FM included in the qualitative synthesis. ACR, American College Of Rheumatology; AID, anti-inflammatory drug; anti-CCP, anti-cyclic citrullinated peptide; AUB, abnormal uterine bleeding; BPI, Brief Pain Inventory [40]; DAS-28, disease activity score -28 [47]; ELISA, enzyme-linked immunosorbent assay; ESR, erythrocyte sedimentation rate; EULAR, European League Against Rheumatism; FIQ, Fibromyalgia Impact Questionnaire [48,49]; FM, fibromyalgia; FOXP3, forkhead box protein P3; GFI, global fatigue index [44]; GI, gastrointestinal; gMFI, geometric mean fluorescence intensity; HADS, Hospital Anxiety and Depression Scale [46]; HAQ-DI, health assessment questionnaire-disability index [42]; HPMC, hydroxy-propyl methyl-cellulose; hs-CRP, high sensitivity C-reactive protein; HRSD, Hamilton Rating Scale for Depression [45]; IFN-γ, interferon-γ; IPAQ, International Physical Activity Questionnaire [50]; LOCF, last-observation carry forward; MDA, malondialdehyde; N/A, not applicable; NR, not reported; NSAIDs, non-steroid anti-inflammatory drugs; ITT, intention-to-treat; OA, osteoarthritis; ONS, oral nutrient supplementation; PE, physical exercise; PGA, Physician Global Assessment [43]; PP, per protocol; PS, Pain score; RA, Rheumatoid arthritis; SJC, swollen joint count; Th, T helper; TJC, tender joint count; TNF-a, tumor necrosis factor α; Treg-cells, regulatory T cells; VAS, visual analogue scale [39]; WOMAC, Western Ontario and McMaster Universities Osteoarthritis Index [41]. * Within the manuscript text, 31 participants were reported to have completed the active arm intervention and 30 controls, in the CONSORT flow chart it appears that 32 women from the intervention and 31 from the control arm were analyzed, but in the tables, the respective number of reported participants in active and comparator arms was 33 and 32. ^‡^ Reported data refer to the PP analysis; ^R^ range; ^M^ mean ± standard deviation.

Details of the RCTs fulfilling the study’s criteria, evaluating saffron interventions in patients with rheumatoid arthritis (RA), osteoarthritis (OA), or fibromyalgia (FM), the respective trials are detailed in Figure 3. The effect of saffron supplementation was evaluated in two trials using participants with RA [27,31,33], an additional two RCTs with patients with a knee OA diagnosis [28,29,32] and finally, on one RCT performed in patients with FM [30] (Figure 3). In RA, two different diagnostic criteria were employed, including the American College of Rheumatology/European league against Rheumatism (ACR/EULAR) 2010 [34] and the revised ACR 2017 [35]. For patients with OA and FB, the ACR [36] and ACR [37] criteria were employed, respectively.

No relevant completed trials were retrieved for spondylarthritis, ankylosing spondylitis, Sjogren’s syndrome, hyperuricemia, systemic lupus erythematosus (SLE), scleroderma, psoriatic arthritis, psoriasis, or Behçet’s syndrome (BS).

#### 3.2.2. Trial Design and Origin

All trials were conducted in Iran and were published between the years 2018 and 2021. The RCTs employed a parallel intervention design. No cross-over trials were retrieved, fulfilling the PICO question of the study. All included RCTs were double blinded [27,28,29,30,32].

#### 3.2.3. Intervention and Comparator Particularities

The administered doses of *Crocus sativus L*. ranged between 15 mg/day [28,30] and 100 mg daily [27,29,31,32,33]. All studies used pills, tablets or capsules for the delivery of saffron supplements. Sahebari and associates [27] used pure saffron powder made of saffron flowers (Saharkhiz Saffron Factory, Mashhad, Iran), Hamidi et al. [31,33] administered saffron Sargol (Saharkhiz Saffron Factory, Mashhad, Iran), and Poursamimi and associates [28] applied interventions with Krocina^TM^ (Samisaz Pharmaceutical Company, Mashhad, Iran). In the Shakiba trial [30], dried and milled *Crocus sativus L*. stigma (IMPIRAN, Tehran, Iran) was used for the preparation of tablets, and Firoozabadi et al. [29,32] administered saffron pills (not-other defined). Extraction information and methods were only provided in two trials [27,30]. Additional compounds in the administered tabs were reported in two trials [27,30], but the exact composition of the final products was not declared in any RCT. Standardization of the final product was only reported by Shakiba and associates [30], based on the crocin and safranal content of the capsules via spectrometry. Although Poursamimi et al. [28] administered ready-to-buy supplements, no information is currently provided on the manufacturer’s website [38]. Intervention duration spanned between 8 weeks [30] and 4 months [28].

Four RCTs used placebos as comparators [27,28,29,31,32,33] and one used duloxetine [30], but the aim in the latter was to assess the comparative effectiveness of saffron versus duloxetine for depression in patients with FM.

#### 3.2.4. Sample Size

The sample size was rather small in all RCTs, spanning from 40 [28] to a maximum of 82 [27] patients per trial, prior to randomization. The included RCTs involved a total of 148 patients with RA, 106 patients with OA, and 54 patients with FM. In the pooled sample, 104 patients received a saffron intervention and 104 were allocated to the control arms. One trial which was only published in abstract format [29,32] did not report the number of patients allocated in the intervention/comparator arms.

### 3.3. Outcomes Assessed in the Included Interventions

#### 3.3.1. Sensation of Pain

One important outcome of interest among the included trials involved pain, which was evaluated using the visual analogue scale (VAS) [39], the pain scale (not defined), the brief pain inventory (BPI) [40], or the Western Ontario and McMaster universities (WOMAC) OA index pain subscale [41].

#### 3.3.2. Immune Response

Immune response post-saffron supplementation was evaluated in one RCT [28] assessing CD8+ and CD4+ T helper (Th) cells, Th17 cell percentage (%), T-regulatory (Treg) cells percentage (%), and the geometric mean fluorescence intensity (gMFI) of forkhead box protein P3 (FOXP3) of Treg cells, as well as the Treg/Th17 ratio.

#### 3.3.3. Inflammation

Assessed inflammation markers included the erythrocyte sedimentation rate (ESR), C-reactive protein (CRP) and hs-CRP (high sensitivity CRP), tumor necrosis factor-α (TNF-α), interferon-γ (IFN-γ), interleukine-17 (ΙL-17), and interleukine-1β (ΙL-1β) levels.

#### 3.3.4. Health Assessment, Depression and Fatigue

Health was self-assessed by the patients themselves using the health assessment questionnaire-disability index (HAQ-DI) [42], or by their physicians using the physician global assessment (PGA) [43]. Fatigue was evaluated using the global fatigue index (GFI) [44] in one trial.

Depression was assessed using Beck’s depression inventory (BDI), the Hamilton depression rating scale [45], or the Hospital Anxiety and Depression Scale (HADS) [46].

#### 3.3.5. Antioxidant Status

Antioxidant activity and oxidative stress were assessed according to the malondialdehyde (MDA) levels, and total antioxidant capacity (TAC) via the ferric reducing ability of plasma (FRAP) method.

#### 3.3.6. Disease-Specific Scores

Disease-specific scores were also evaluated, depending on the diagnosis of the participants in each trial. For RA, the disease-specific scores involved the disease activity score-28 (DAS-28) including the ESR assay [47] (DAS-28-ESR) and the swollen and tender joint count (SJC, TJC). For the RCT performed in patients with FM [30], the fibromyalgia impact questionnaire (FIQ) [48,49] was applied. In the case of OA, one trial [29,32] reported using the WOMAC [41].

### 3.4. Risk of Bias Summary

**Figure 4:**
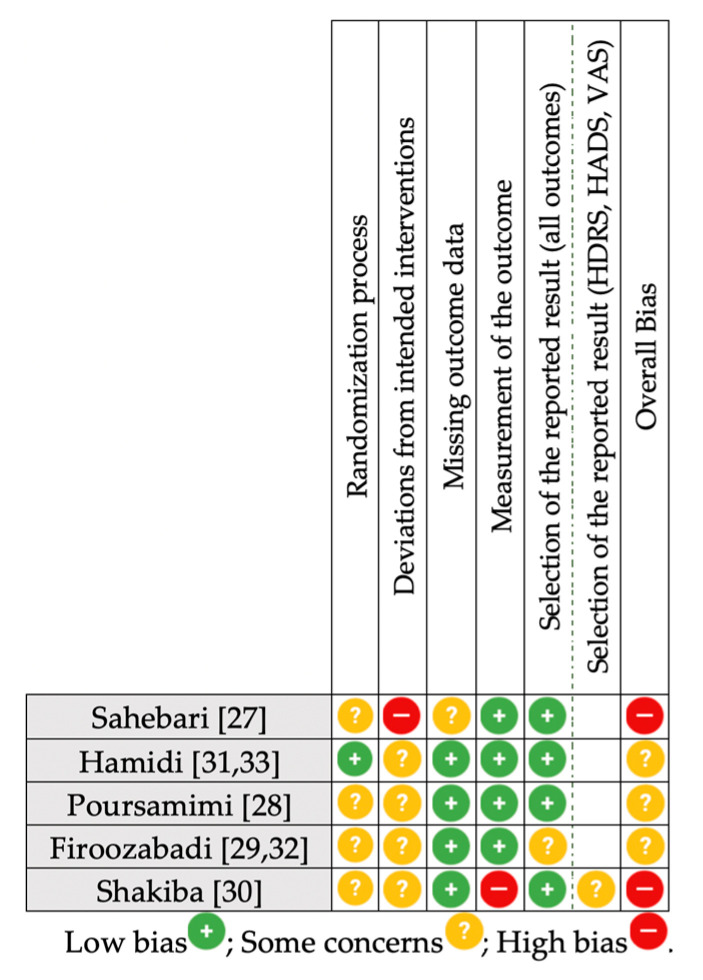
Summary of risk of bias [24] assessment for the included RCTs. HADS, Hospital Anxiety and Depression Scale; HDRS, Hamilton Depression Rating Scale; RCT, randomized controlled trial; VAS, visual analogue scale.

The summary of risk of bias for the included RCTs is presented in Figure 4. The majority of RCTs (60%) exhibited some concerns for overall risk of bias, with the remaining 40% having a high risk for overall bias. The greatest proportion of trials with unclear bias involved the randomization process and the deviations from intended interventions domains.

### 3.5. Other Bias

#### 3.5.1. Treatment Adherence

Treatment adherence was assessed only in two trials [30,31,33], with the remaining studies failing to control for this issue. Furthermore, the ban of antioxidant supplements at the beginning of the trials was not reported by any trialist, whereas in Shakiba’s trial [30], only a history of treatment with saffron was an exclusion criterion, without controlling for other antioxidants.

#### 3.5.2. Dietary Intake and Exercise Patterns

Diet was only recorded and assessed by Hamidi et al. [31,33], despite the fact that it can alter antioxidant intake. Similarly, physical activity a known mediator of disease activity and stress was only assessed by Hamidi [31,33].

### 3.6. Adherence to the CONSORT Statement for RCTs with Herbal Medicine

**Figure 5:**
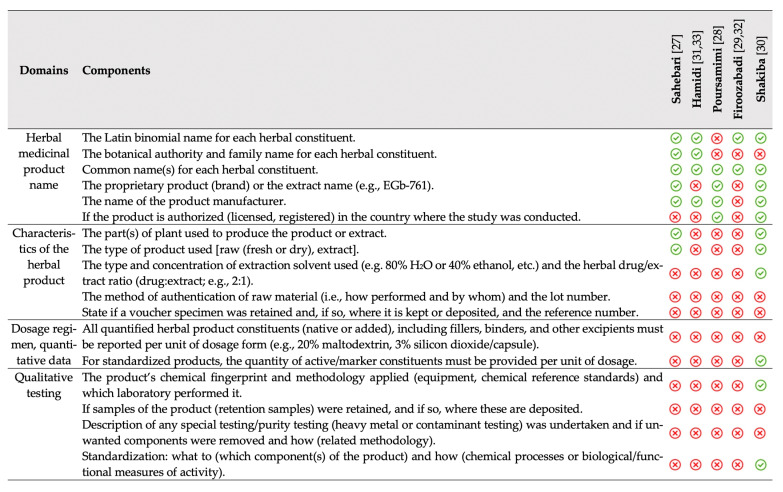
Summary of adherence to the formula elaborations suggested by the CONSORT statement for RCTs implementing herbal medicine interventions [17]. CONSORT, Consolidated Standards of Reporting Trials; RCT, randomized controlled trial. 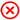 not reported; 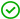 reported.

Among the included trials, the majority failed to adhere to the formula elaborations suggested by the Consolidated Standards of Reporting Trials (CONSORT) statement for RCTs including herbal medicine interventions [17] (Figure 5). Thus, it appears that the exact composition and dosage of active saffron ingredients, including crocin, crocetin or safranal, cannot be calculated, with the exception of one trial [30]. Shakiba’s RCT [30] adhered to the majority of CONSORT components involving the standardization and procedures required for RCTs with herbal medicine interventions. On the other hand, Sahebari [27] also reported all added constituents, but failed to define the exact dosage per administered unit. Firoozabadi and associates [29,32] demonstrated the least adherence; however, their results were only published in abstract format, and thus limited space was available.

### 3.7. Results

Regarding the sensation of pain, ONS with *Crocus sativus L.* either reduced [27,28,33], or did not appear to have an effect [27,29,30,32] when administered to patients with RA, OA or FM. The use of NSAIDs was reduced in one trial using a sample of patients with OA [29,32]. On the other hand, no change was recorded regarding the sensation of fatigue in FM (one trial) [30].

Markers of inflammation were examined in RA and OA and were either reduced or remained unchanged post-intervention, with trials indicating conflicting results. Indicators of antioxidant activity and oxidative damage remained unchanged (MDA [31,33] and TAC [31,33] in one RCT each), raising concerns regarding the efficacy of saffron.

Immune response was evaluated in one OA RCT [28], which reported an increase in the percentage of Treg-cells, the Treg/Th17 ratio and a decrease in the Th17 cell percentage.

In RA, disease-specific indexes such as the DAS-28-ESR, morning stiffness, TJC and SJC were reduced in one RCT [31,33] and were not affected in another [27]. In OA, results concerning the total WOMAC score were not reported by the trialists [29,32]. In FM, saffron ONS did not affect the FIQ among participants [30].

Depression scores remained unchanged in individual trials in patients with RA [27] and FM [30]. ONS with saffron failed to induce improvement in physical function among patients with RA (one RCT) [27]. Last, self-rated health assessment remained unchanged in RA post-intervention [27], but was improved when assessed by the physicians using the PGA in patients with RA [31,33].

### 3.8. Adverse Events

In the present systematic review, two [28,29,32] out of five RCTs failed to report adverse events. The most frequently reported issues following saffron supplementation involved xerostomia, abdominal pain, vomiting, anxiety, palpitations, etc.

### 3.9. Synthesis without Meta-Analysis (SWiM)

**Figure 6:**
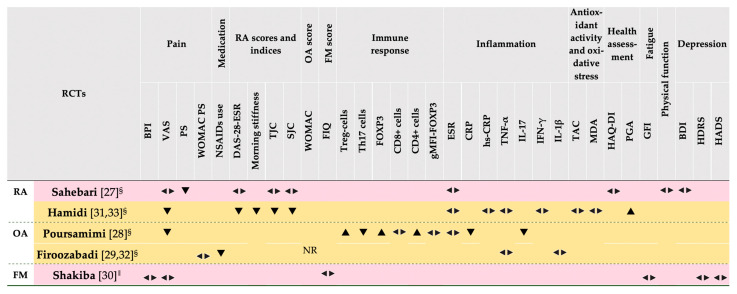
Qualitative synthesis without meta-analysis of the outcomes in each RCT, favoring the saffron arms post-intervention. All RCTs had less than 50 participants in each arm. Row background colors denote study quality according to the RoB assessment. BDI, Beck Depression Inventory [51]; BPI, Brief Pain Inventory [40]; DAS-28, disease activity score 28 [47]; ESR, erythrocyte sedimentation rate; FIQ, Fibromyalgia Impact Questionnaire [48,49]; FM, fibromyalgia; FOXP3, forkhead box protein P3; GFI, global fatigue index [44]; gMFI, geometric mean fluorescence intensity; HADS, Hospital Anxiety and Depression Scale [46]; HAQ-DI, health assessment questionnaire-disability index [42]; HDRS, Hamilton Depression Rating Scale [45]; hs-CRP, high sensitivity C-reactive protein; IFN-γ, interferon-γ; MDA, malondialdehyde; NR, not reported; NSAIDs, non-steroid anti-inflammatory drugs; OA, osteoarthritis; PGA, Physician Global Assessment [43]; RA, rheumatoid arthritis; RCT, randomized controlled trial; RoB, risk of bias [24]; SJC, swollen joint count; TAC, total antioxidant capacity; Th, T helper; TJC, tender joint count; Treg, T regulatory; ^‖^ intervention duration <3 months; ^§^ intervention duration between 3–4 months; ▲ increased; ▼ reduced; 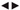 no change.

Figure 6 details the effect direction plot of the outcomes assessed in the included RCTs. For the majority of outcomes, conflicting results are apparent. Moreover, for most outcomes, less than three RCTs have provided results regarding similar outcomes.

For the outcome of pain (VAS), four RCTs provided results, with two indicating a reduction in pain and two failing to reveal an effect. Regarding the levels of ESR, and TNF-α, three and two of the included RCTs, respectively, provided results. However, for both ESR and TNF-α, the direction plot was similar in all, indicating a lack of effect following saffron supplementation in patients with RA and OA.

Due to the heterogeneity of the RCTs and the lack of data regarding the standardization of the herbal medicine interventions, a meta-analysis was not deemed as a safe option.

## 4. Discussion

The present SWiM assessed the effects of supplementary *Crocus sativus L.* intake on disease-related outcomes among patients with a RD diagnosis. It appears that limited RCTs have been performed on this issue, thus demonstrating1 that the evidence is not enough to secure a positive direction of effect for any of the examined outcomes. Moreover, serious pitfalls regarding the reporting of the intervention formulas are apparent, further reducing the quality of the trials.

Consumption of saffron can reduce inflammation through inhibition of the cyclooxygenase enzyme activity [52]. According to a recent meta-analysis [9], saffron is effective in improving the levels of inflammatory markers such as TNF-α, IL-6 and CRP when administered at specific doses (≤30 mg/day) in young adults (<50 years old) lacking a diabetes diagnosis. In the present review, only four trials administered a dose not exceeding 30 mg/day [28,30], with only one [28] evaluating inflammatory markers among participants. Interestingly, CRP and IL-17 were improved in this trial post-intervention. Thus, it is possible that the higher doses administered in the rest of the trials [29,31,32,33] might have produced a negative or null effect. Nevertheless, another meta-analysis [9] failed to detect any differences regarding CRP, TNF-α, and IL-6 between the saffron and placebo arms. These discrepancies, however, may lay in the underline pathologies of the participants, the duration of interventions, or differences in the standardization of the administered supplements.

Research indicates that saffron can reduce the concentrations of endogenously generated reactive oxygen species, inhibiting oxidative damage, while reducing the production of pro-inflammatory biomarkers [9,53]. According to a recent meta-analysis [54], supplementation can induce improvements in the MDA and TAC levels. However, no improvements were revealed in the present SWiM, due to the small number of studies evaluating these outcomes, most of which indicated a null effect.

Depression and anxiety are common problems in patients with chronic disease and rheumatic disease in particular. Moreover, recent meta-analyses indicate that ONS with *Crocus sativus L.* may improve depressive symptoms and anxiety [12,55]. This effect is persistent even when used as an adjunct to antidepressants, as in the present RCTs. Moreover, specific depression batteries such as the BDI appear to be more sensitive to saffron ONS, whereas the HDRS has been reported to be less flexible [56]. Saffron has been suggested to entail relaxant, inhibitory effects on both histamine (H1) and the muscarinic receptors [57]. By inducing relaxation and reducing anxiety, supplementation with *Crocus sativus* can also improve sleep quality [58]. On the other hand, improved sleep is associated with less fatigue. Overall, previous evidence synthesis indicates that saffron is more efficient compared to placebo and additionally equally effective with synthetic antidepressants [59,60]. These findings, however, were not akin to the present SWiM due to the probable methodological pitfalls of the included trials, heterogeneity and lack of information regarding the standardization of the intervention formulations.

Regarding pain, no meta-analyses have evaluated the effect of saffron ONS, although individual RCTs performed on patients with distinct diagnoses indicate possible improvement in the sensation of pain [61].

According to research, the dried stigmas and tops of the plant styles have the majority of medicinal properties, including immunomodulating responses. Saffron contains a variety of mineral agents, glycosides, anthocyanins, alkaloids, carotenoids and flavonoids including quercetin and kaempferol, which further increase its immunoregulatory properties [62,63]. Studies using animal models have revealed that saffron acts on selective Th2 upregulation, naming it a “nutraceutical” spice [64]. Other preclinical and animal studies showed that saffron can increase the expression levels of FOXP3, a transcriptional factor, in Treg cells, and suppress IL-10 and IFN-γ secretion [65,66,67]. In the present SWiM, only one trial [28] evaluated immune response post-saffron supplementation, indicating improved immunomodulation. However, further studies are required, assessing similar outcomes.

### 4.1. Methodological Limitations of the Included Trials

#### 4.1.1. Assessment of Treatment Adherence Rate

According to research, treatment adherence in clinical trials is suboptimal, affecting the economic costs, while impacting the methodological quality of the trials [68]. Nearly half of the RCTs involving oral pharmacological interventions failed to report adherence rates [69], indicating that proper adherence consideration is the exception instead of the rule [68]. In the present review 40% (*n* = 2) of the included RCTs reported assessing treatment adherence, although the exact rates were not presented. Moreover, none of the trials adhered to the ESPACOMP Medication Adherence Reporting Guideline (EMERGE) reporting guidelines regarding treatment adherence assessment [68]. A high non-adherence rate can reduce a trial’s ability to detect a true treatment effect [70]. If adherence was considered and reported, the results regarding *Crocus sativus L.* supplementation in RDs might have been different.

#### 4.1.2. Possible Cross-Treatment Effect

The standard treatment of participants was not reported in all trials. In the Sahebari et al. RCT [27], vitamin D ONS was among the standard therapy received by the participants and changes the sensation of pain was one of the outcomes of interest. Although pain was improved post-saffron administration [27], the scientific literature indicates that vitamin D might influence immune cells and pain sensitization through a variety of hormonal and neurological pathways [71,72]. Thus, the improved pain sensation noted in the trial might well be the synergistic result of vitamin D and *Crocus sativus L*.

Similarly, in the trial conducted by Poursamimi and associates [28], as the authors promptly noted, the improved pain relief observed may be the result of sodium diclofenac, which was administered to all participants during the trial. For this, significant improvements regarding pain were noted in both arms [28].

#### 4.1.3. Differentiation between OA and RA

In the present SWiM, RCTs performed in patients with an OA diagnosis were also included, as often, patients with RA are misdiagnosed with OA, and vice versa [22]. Thus, it is possible that some of the patients included in the trials might have belonged in the opposite diagnosis, despite recruitment intentions.

Among the included RCTs, the one conducted by Sahebari and associates [27] was the only one where the anti-cyclic citrullinated peptide (anti-CCP)-positive patients were assessed within the sample, reporting that 89.2% of those allocated in the intervention and 81.6% of the controls were positive. The remaining RA/OA trials [28,29,31,32,33] failed to address this issue. Since this is a common problem in arthritis research, including both diagnosis without merging them was deemed as the safest option for the SWiM.

#### 4.1.4. Effect of Lifestyle on RD Outcomes

Lifestyle has an impact on disease activity and outcomes in patients with RDs. In further detail, exercise can reduce disease activity and diet can either improve or amplify symptoms related to the diseases [73,74,75,76,77]. For this, the diet of participants in each RCT with ONS interventions must be recorded, and in parallel, physical activity should also be monitored. Among the included RCTs, however, only one [31,33] evaluated the diet of participants and their physical activity levels. The remaining failed to control for this important factor, introducing bias to their results.

#### 4.1.5. Standardization of the Herbal Intervention and Reporting Quality of Formula Elaborations

As Ali [78] noted, herbal medicines tend to suffer from lack of standardization parameters. In more detail, there appears to be a lack of standardization regarding the raw materials used, the harvesting, drying, storage and processing methods, as well as the final products and dosage formulation [16,79]. Moreover, quality control procedures are inexistent in most of the trials [79]. According to the World Health Organization (WHO), all medicines, whether they are of plant origin or synthetic, must fulfill the basic requirements of safety and effectiveness [16,80]. Nevertheless, it appears that trials implementing herbal medicine interventions often fail to report information required to judge internal validity, external validity, and reproducibility [17,18]. From the bush to the content of a pill, herbal substances undergo a variety of procedures that define the final product’s active ingredients and may greatly affect efficacy. As a result, most frequently, batch-to-batch uniformity of the active constituents and quality control using various analytical techniques are inexistent [81], leading to substantial variations in the formulation and bioactivity of herbal medicine supplements from lot to lot [82], and it is unclear if single and consistent batches are used for the formulations applied in the trials. Moreover, the need to quantify the test substance using high-performance liquid chromatography, gas chromatography, or other techniques is required to understand the exact dose of active ingredient that produces a significant effect [81].

According to Guo [83], the often non-standardized nature of the prepared interventions increases the probability for adverse events, indicating that in all cases of RCTs with herbal medicine, standards of safety and efficacy must be implemented. Today, poor reporting of adverse events consists of a frequent criticism regarding CAM research [84,85] and in the present systematic review 2/3 of the RCTs failed to report any adverse reactions. Moreover, serious adverse events have been reported by the FDA; however, as they are rare, they often fail to be manifested in small or underpowered RCTs [82].

Apart from the CONSORT for herbal medicine interventions [17], a variety of additional guidelines have been published with regard to quality standards and good clinical practice in herbal medicine trials, including WHO recommendations and International Union of Pure and Applied Chemistry (IUPAC) protocols [86,87,88,89]. Furthermore, information regarding fingerprinting analyses for the quality assessment of herbal medicine have also been proposed for interested stakeholders [90].

In the present systematic review, it was shown that regarding RCTs with saffron interventions in patients with RDs, the majority failed to adhere to the CONSORT-specific requirements for herbal medicine interventions. Similar issues have also been reported to exist in Cochrane systematic reviews evaluating herbal medicine [91]. For this important limitation, despite the plethora of meta-research evaluating herbal medicine interventions that have been published in high-end academic journals without considering this limitation [84,92], we considered that any quantitative synthesis would be misleading for the authors and clinical practice, and was avoided.

#### 4.1.6. Intervention Duration

The duration of the intervention varied greatly in the included RCTs, spanning from as low as 8 weeks [30] to 4 months [28]. It is possible that a longer intervention duration might have changed the results in several trials, as other trials administering saffron for other conditions have, in their majority, applied the interventions for 3–4 months [9,15,93], with a respective follow-up session. Moreover, according to a recent meta-analysis, longer saffron supplementation durations have been shown to improve outcomes with regard to blood pressure [15]. Suffice to say, the exact intervention duration required to produce beneficial effects for each outcome has not yet been delineated.

#### 4.1.7. Country of Origin

All trials included in the present SWiM were conducted in Iran. Today, 80% of the global saffron production is harvested from Iran, and this is why Iranian researchers are keen on investigating the plant’s properties [57]. Nevertheless, according to an umbrella review [94], when studying the available literature, the need to conduct higher-quality trials outside of Iran becomes apparent, in order to reduce bias.

### 4.2. Ongoing Trials

**Figure 7:**
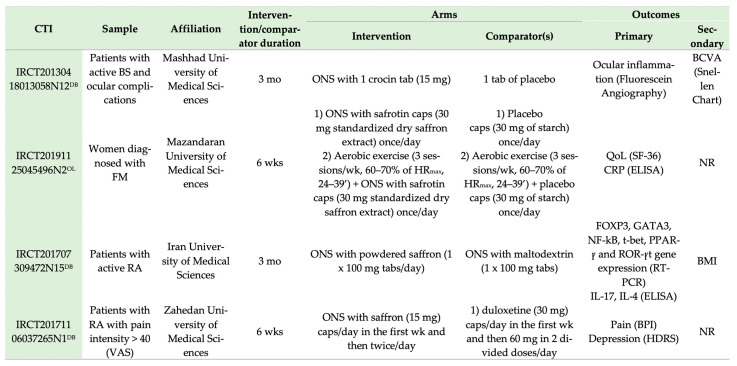
Parallel RCTs investigating ONS with saffron in patients with rheumatic diseases. BCVA, best corrected visual acuity; BMI, body mass index; BPI, brief pain inventory; BS, Behcet’s syndrome; CRP, C-reactive-protein; CTI, clinical trial identifier; ELISA, enzyme-linked immunosorbent assay; FM, fibromyalgia; FOΧP3, forkhead box P3; HDRS, Hamilton Depression Survey Questionnaire; HR_max_, maximum heart rate; IL-4, interleukin-4; IL-17, interleukin-17; mo, months; NF-kB, nuclear factor kappa-light-chain-enhancer of activated B cells; NR, not reported; ONS, oral nutrient supplementation; PPAR-γ, peroxisome proliferator-activated receptors γ; QoL, quality of life; RA, rheumatoid arthritis; RCT, randomized controlled trial; ROR-γt, RAR-related orphan receptors γt (thymus-specific isoform); RT-PCR, real-time polymerase chain reaction; SF-36, Short Form 36; T-bet, T-Box protein expressed in T cells; VAS, visual analogue scale; wks, weeks; ^DB^ double blind; ^OL^ open label.

Figure 7 details the ongoing trials investigating the effect of saffron in patients with RDs. A total of four RCTs were identified in the Iranian registry of clinical trials (IRCT) and none in the clinicaltrials.gov database. These trials are recruiting patients with BS, RA or FM, investigating similar outcomes as in the present review. Their results are expected to aid in understanding the possible results of saffron supplementation among patients with rheumatic diseases.

### 4.3. Limitations of the Present Qualitative Synthesis

The limitations of the present qualitative synthesis primarily involve the lack of an adequate number of trials investigating similar outcomes in distinct RDs. Furthermore, a gap in the literature is apparent, with null saffron RCTs conducted for specific RDs (psoriasis, SLE, ankylosing spondylitis, Sjogren’s syndrome, etc.)

As in every meta-research, the present review also carries the limitations of the included trials, indicating that there is room for the methodological improvement of RCTs investigating saffron in RDs. Interestingly, most of the included trials failed to assess and report changes in disease-activity specific scores (e.g., WOMAC), an issue that should be accounted for when designing future trials. Moreover, the high clinical and methodological heterogeneity among the included trials did not allow for a meta-analysis to be performed. According to a recent umbrella systematic review [94], RCTs evaluating saffron interventions entail a variety of biases, and their methodology should be improved.

The need for evaluating herbal medicine interventions is indisputable. Today, it is estimated that 2/3 of the global population uses herbal medicines, with some countries having incorporated them into the public health system [88]. Nevertheless, serious doubts regarding their safety and effectiveness remain [95]. According to Ernst and Pittler [96], the majority of studies published in CAM journals report positive findings and the concerns regarding the variation in formulation and bioactivity of some supplements remain a challenge [82]. As suggested by the European research network for CAM [97], CAM constitutes a neglected research area requiring more activities; however, specific standards of reporting must be met in advance. Although the assessment of the adherence to the CONSORT guidelines for the conduction and reporting of herbal medicine RCTs was not included in the initial aims of the present systematic review or the protocol, during the peer review process, it became clear that this issue constitutes an important factor affecting trial quality and intervention efficacy. This additional analysis added value to the present review, highlighting an area in need of improvement regarding the reporting of these trials.

## 5. Conclusions

Pedanio Dioscorides, an ancient Greek medical practitioner, was the first to report the medicinal properties of saffron [52,57]. In an extensive review of the history and the literature, Christodoulou [57] underlined the value of saffron over the centuries, with the “Saffron war” taking place in the Middle Ages and the execution of those who dared to tamper with saffron’s composition due to its medicinal properties. Today, in the era of evidence-based medicine, whether this value can also be evidence-based greatly depends on the appraisal of the existing primary studies.

Research has suggested that *Crocus sativus* can form an effective adjuvant therapy for many conditions, and a promising one for RDs. RCTs performed in patients with RDs indicate that saffron may target many different outcomes, including inflammation, antioxidant status, depression and anxiety, pain, immune response and many others. If its efficacy is demonstrated, then it will undoubtedly be the “golden spice” for RDs. Nevertheless, at the moment, more primary studies are required to help us find the appropriate dose and conclude with certainty on the efficacy of saffron ONS in rheumatic diseases-related outcomes. Furthermore, the present systematic review raised concerns regarding the importance of reporting standards in herbal medicine research, with chemical fingerprinting being a required prerequisite for the standardization, safety and efficacy evaluation of the active ingredients.

## Figures and Tables

**Table 1:** PICO components of the study’s research question.

**P**opulation	Patients with any rheumatic disease diagnosis
**I**ntervention	Saffron (tabs, sachets, pills, tea, etc.)
**C**omparison	Placebo, or any other intervention
**O**utcomes	Any disease-specific (immediate/intermediate) or comorbidity-related outcome

## Data Availability

All data are presented within the manuscript text.
